# Intraoperative Evaluation of Soft Tissue Sarcoma Surgical Margins with Indocyanine Green Fluorescence Imaging

**DOI:** 10.3390/cancers15030582

**Published:** 2023-01-18

**Authors:** Matthew F. Gong, William T. Li, Sumail Bhogal, Brittany Royes, Tanya Heim, Maria Silvaggio, Marcus Malek, Rajeev Dhupar, Stella J. Lee, Richard L. McGough, Kurt R. Weiss

**Affiliations:** 1 Department of Orthopaedic Surgery, University of Pittsburgh Medical Center, Pittsburgh, PA 15232, USA; 2 Department of Pediatric General and Thoracic Surgery, Children’s Hospital of Pittsburgh, Pittsburgh, PA 15224, USA; 3 Department of Thoracic Surgery, University of Pittsburgh Medical Center, Pittsburgh, PA 15232, USA

**Keywords:** sarcoma, soft tissue sarcoma, indocyanine green, musculoskeletal oncology, tumor margin

## Abstract

**Simple Summary:**

Soft tissue sarcomas (STS) are rare malignant tumors associated with poor outcomes and high local recurrence rates. Current tools available to the STS surgeon for intraoperative and definitive margin assessment include intraoperative frozen section and permanent pathology, respectively. Indocyanine green (ICG) is a historically safe fluorophore dye that has demonstrated efficacy for intraoperative margin assessment in both breast and gastrointestinal cancers. This study presents an ongoing, prospective, non-randomized clinical study implementing ICG intraoperative margin assessment in patients with confirmed or suspected STS. Eighteen STS cases have been enrolled at the time of this writing. ICG margins were found to match permanent pathology margins in 56% (10/18) of patients, with 22% sensitivity and 89% specificity. ICG may be promising for STS intraoperative margin assessment, although larger sample sizes are necessary to better quantify optimal dosage, timing, the effect of histological subtype, and the ultimate capacity of this technology to decrease STS local recurrence.

**Abstract:**

Soft tissue sarcomas (STS) are rare malignant tumors often associated with poor outcomes and high local recurrence rates. Current tools for intraoperative and definitive margin assessment include intraoperative frozen section and permanent pathology, respectively. Indocyanine green dye (ICG) is a historically safe fluorophore dye that has demonstrated efficacy for intraoperative margin assessment in the surgical management of both breast and gastrointestinal cancers. The utility of ICG in the surgical management of sarcoma surgery has primarily been studied in pre-clinical mouse models and warrants further investigation as a potential adjunct to achieving negative margins. This study is a prospective, non-randomized clinical study conducted on patients with confirmed or suspected STS. Patients younger than 18 years, with a prior adverse reaction to iodine or fluorescein, or with renal disease were excluded from the study. Intravenous ICG was infused approximately three hours prior to surgery at a dosage of 2.0–2.5 mg/kg, and following tumor resection, the excised tumor and tumor bed were imaged for fluorescence intensity. When scanning the tumor bed, a threshold of 77% calibrated to the region of maximum intensity in the resected tumor was defined as a positive ICG margin, according to published protocols from the breast cancer literature. ICG results were then compared with the surgeon’s clinical impression of margin status and permanent pathology results. Out of 26 subjects recruited for the original study, 18 soft tissue sarcomas (STS) were included for analysis. Three subjects were excluded for having bone sarcomas, and five subjects were excluded due to final pathology, which was ultimately inconsistent with sarcoma. The average age of patients was 64.1 years old (range: 28–83), with an average ICG dose of 201.8 mg. In 56% (10/18) of patients, ICG margins were consistent with the permanent pathology margins, with 89% specificity. The use of ICG as an intraoperative adjunct to obtaining negative margins in soft tissue sarcoma surgery is promising. However, studies with larger sample sizes are warranted to further delineate the accuracy, optimal dosage, timing, and types of sarcoma in which this diagnostic tool may be most useful.

## 1. Introduction

Sarcomas are rare and aggressive mesenchymal tumors that comprise ~1% of adult malignancies and 7–15% of pediatric malignancies [1,2,3]. Around 60–80% of sarcomas originate from the soft tissues of the extremities, and more than 50 different histological subtypes of these soft tissue sarcomas (STS) have been identified [1,2]. In 2022, the National Comprehensive Cancer Network (NCCN) predicted that approximately 13,190 people would be diagnosed with STS, with around 5130 estimated deaths [1]. Surgical resection is the primary treatment of STS, with neoadjuvant or adjuvant radiotherapy and chemotherapy serving as important adjunct treatments on a case-by-case basis. Prior studies have reported local recurrence (LR) rates between 10–30%, with some studies noting LR rates as high as 40% [4,5,6,7,8,9,10,11]. Obtaining negative margins on final pathology following surgical resection is a key predictor of LR, with histologic grade, tumor size, and tumor depth also being important risk predictors of LR and metastasis [7,12]. Currently, the surgeon’s clinical impression is the most common means of intraoperative margin assessment. Frozen section analysis is used sporadically, and final pathology is considered the most definitive assessment of margin adequacy. In certain circumstances, planned positive margins may be left intentionally at the discretion of the surgeon. Common reasons for planned positive margins include if a margin is acceptably close to a critical neurovascular structure or if the pre-operative goal of the surgery was palliative tumor debulking. When positive margins occur, adjuvant therapy can help improve local control to reduce the likelihood of LR [13,14]. Positive margins which occur unexpectedly may also warrant a return to the operating room for further surgical resection. LR can result in devastating consequences, including an amputation rate ranging from 10–20%. Patients who experience LR have a statistically greater likelihood of metastatic disease and death compared with patients where initial local control is successfully achieved [13,15,16]. Clearly, the importance of achieving a negative margin during STS surgery cannot be understated, and the high rate of LR is a major limitation of sarcoma care. Better intraoperative margin assessment tools would be an invaluable adjunct in the hands of the sarcoma surgeon. Intraoperative indocyanine green (ICG) fluorescence imaging may have the potential to reduce LR rates in STS patients.

Various techniques utilizing fluorescent imaging are under ongoing investigation for intraoperative soft tissue sarcoma margin assessment. These have included ICG fluorescence imaging as well as imaging via antibody-fluorophore conjugates such as bevacizumab-800CW (targeted to VEGF-A) and ABY-029 (targeted to EGFR) [17,18]. ICG fluorescence imaging emerged as a technique utilized to visualize abnormal tumor tissue and aid in achieving a resection with negative margins in the treatment of various breast, thoracic, abdominal, and urologic cancers [19,20,21,22,23]. ICG itself is an FDA-approved tricarbocyanine fluorescent dye with an excellent historical safety profile. When administered, ICG has been observed to be safe in nearly all individuals. In very rare circumstances, cases of anaphylactic shock have been observed in patients receiving ICG, with increased risk amongst subjects with a prior iodine contrast allergy [21,24]. Typically, the technique begins with an infusion of ICG prior to surgery and the use of a near-infrared camera to visualize and quantify fluorescence in the intraoperative setting. Although the exact mechanism of tumor uptake of ICG remains unknown, Onda et al. found preferential tumor uptake of ICG occurs via clathrin-mediated endocytosis—whereas ICG is normally cleared rapidly in normal tissue [25]. Chan et al. also noted the effect of ICG pooling near regions of aberrant architecture due to the enhanced permeability and retention (EPR) effect [26]. In prior studies, ICG infusion has been administered at doses ranging from 0.25 to 5 mg/kg at several different time points, including one day prior to surgery, on the day of surgery, at the time of anesthesia induction, or intraoperatively [22,23,27,28]. The work of Pop and Veys in the breast cancer literature is foundational. According to their protocol, the detection of a positive margin has been predicated on comparing fluorescence emitted from the brightest part of the resected tumor (calibrated to 100%) compared with the resected wound bed, with a fluorescence of ≥77% detected in the wound bed identifying a positive margin [29,30]. Implementation of ICG to visualize STS has been validated in pre-clinical mouse models [31,32], and clinical studies have begun to explore its efficacy with a few recently published case series [27,28]. The use of ICG has also been studied in the setting of surgical management of STS to predict the risk of post-operative wound complications such as wound dehiscence or infection [33]. The implementation of similar principles of fluorescent ICG imaging in the treatment of other malignancies may be applied to improve STS resection performance.

The objective of our ongoing study is (1) to assess the feasibility of implementing ICG fluorescence imaging to visualize STS tumors intraoperatively and (2) to compare this technique to surgeon impressions of margin adequacy and final pathology reports. We hypothesize that ICG can be used to effectively visualize the margins of STSs and accurately indicate areas where margins are positive or negative and may thus augment the eyes and hands of an experienced STS surgeon. Based on this, we hypothesize that ICG fluorescence imaging may ultimately assist surgeons to achieve more reliably negative margins and may, in turn, decrease the incidence of LR.

## 2. Materials and Methods

### 2.1. Study Design

This study is a prospective, observational, non-randomized clinical study conducted on patients at a single center with a biopsy of suspicious soft tissue or bone sarcomas between 2021 and 2022. The University of Pittsburgh IRB approval (IRB Study #19110051) and registration with ClinicalTrials.gov (#NCT04719156, accessed on 22 January 2021) were obtained. Inclusion criteria included patients with imaging and/or biopsy results concerning soft tissue or bone sarcoma (both primary or recurrent) undergoing surgical resection. Exclusion criteria included patients younger than 18 years and any patients with a history of an adverse reaction to iodine/fluorescein or renal disease. Informed consent was obtained from patients prior to conducting any study-related procedures, and patients were able to withdraw participation from the study at any time.

### 2.2. Study Protocol

**Figure A1:**
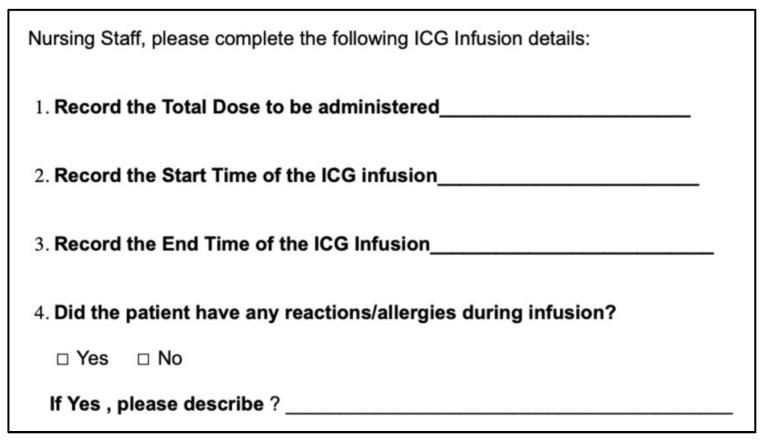
Pre-operative Infusion Form—This form was provided to and completed by the pre-operative clinical staff during the ICG infusion.

Patients presenting for surgical resection for a confirmed or suspected STS were screened for participation in the study. Patients who passed the inclusion and exclusion criteria were then contacted and approached prior to their surgical date. On the patient’s day of surgery, patients arrived at least 3 h prior to their scheduled surgical start time. ICG dosed by weight (2.0–2.5 mg/kg) was prepared in a sterile water dilution by the hospital’s investigational drug pharmacy and subsequently infused intravenously by pre-operative clinical staff. Due to the relatively larger dosage administered, an infusion was performed over an intended duration of 45 min per the instructions of our investigational pharmacy. During the infusion period, patients were monitored by clinical staff and study personnel for any potential infusion reactions. The infusion start and end times were recorded for each patient (Figure A1).

**Figure 1:**
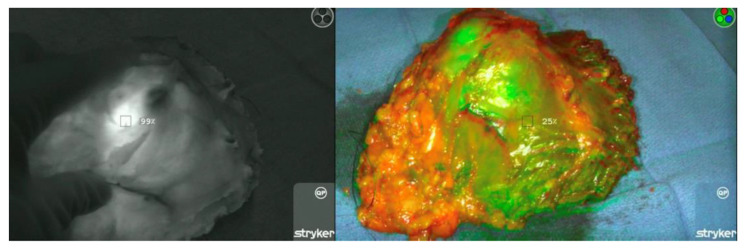
Calibration being performed to the area of highest fluorescence in the resected tumor. Under different camera filter modalities, the SPY-PHI camera is calibrated. Left—Calibration of the region of highest fluorescence to the baseline of 100%. Right—Scanning the remainder of the tumor to ensure no regions exceed calibrated 100% ceiling.

The patient then underwent the surgical procedure as planned. Study personnel comprised of resident physicians recorded the operation’s time of incision, and the surgical team proceeded with the operation until the tumor resection was completed. When the resected tumor was placed on the sterile back table, a member of the study personnel then entered the surgical field and set up a sterilely draped Stryker SPY-PHI near-infrared camera system (Stryker Endoscopy, Kalamazoo, MI, USA). The SPY-PHI camera reports measurements on a calibrated percentage scale, and the area of the highest fluorescence intensity detected in the resected tumor was calibrated to 100% using the SPY-PHI software (Figure 1).

**Figure 2:**
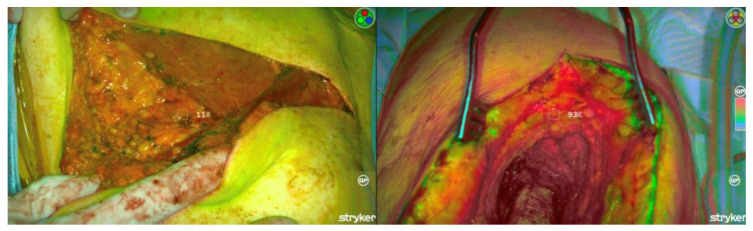
Scanning of tumor bed to assess fluorescence emitted from margins. Left—A tumor bed with fluorescence detected below the 77% threshold, suggesting a negative margin. Right—Tumor bed with fluorescence detected ≥77%, suggesting a positive margin.

In order to optimize the detection of fluorescence, lights in the operating room were dimmed, and the SPY-PHI camera was held 1–2 feet from the area of interest (resected tumor or tumor bed) to obtain a consistent measurement of fluorescence intensity. Of note, the calibration of tumor fluorescence intensity was measured with the normal tissue margin maintained intact without further exposure to the resected tumor’s interior. After calibration, the tumor bed was completely scanned using the camera, with a positive ICG margin defined as any area where fluorescence intensity was ≥77% of the calibrated maximum tumor intensity (Figure 2).

**Figure 3:**
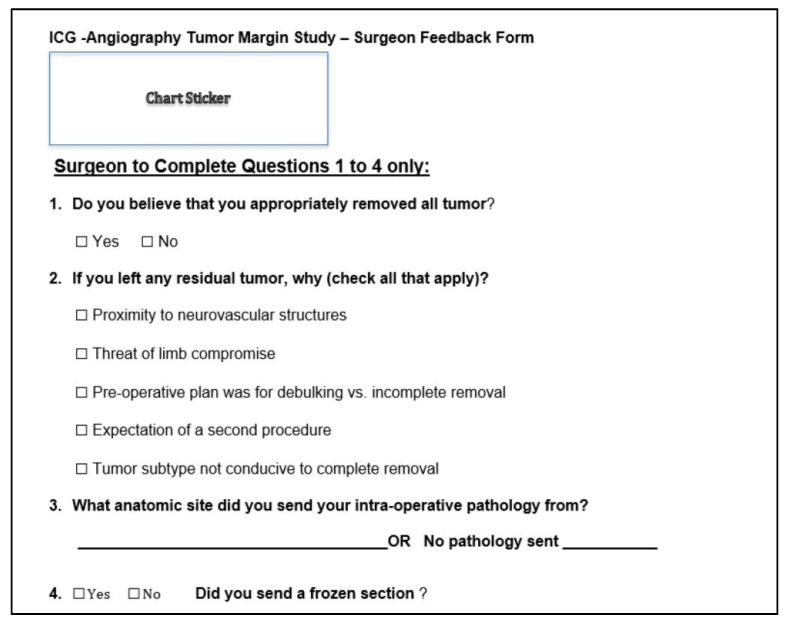
Intraoperative survey where surgeon feedback was recorded on if they believed negative margins were obtained. The survey was completed with surgeons blinded to the results obtained from intraoperative ICG fluorescent imaging.

After the imaging concluded, the attending surgeon, who was blinded to the ICG results, completed an intraoperative feedback survey (Figure 3) on their assessment of the case’s margin status. This intraoperative survey also evaluated if the surgeon intentionally left any residual tumor and the reasons why this was done. The resected tumor was then delivered to pathology, and the final pathology report was reviewed retrospectively in each patient’s electronic medical record to determine if positive or negative margins were present. Retrospectively, each of the intraoperative videos recorded for each patient was reviewed to measure tumor-to-background ratio (TBR), corresponding to the percentage of fluorescence in grossly appearing normal tissue taken as a ratio to the most fluorescent region of the resected tumor (TBR = % Fluorescence of Tumor/% Fluorescence of Normal Tissue).

### 2.3. Statistics

Demographic information, infusion time, time from infusion to initial surgical incision, and STS histologic subtype for each of the participating subjects were reported. Final pathology was considered the standard of care for calculating the accuracy, sensitivity, specificity, positive predictive value (PPV), and negative predictive value (NPV) of our ICG margin assessment technique. Comparisons were then made between cases where ICG results and final pathology results were congruent versus incongruent using a Student’s *t*-test (a = 0.05) for continuous variables and Fischer’s exact test (a = 0.05) for categorical variables. All statistical analyses were performed in GraphPad Prism version 9.4.1.

## 3. Results

### 3.1. Demographics

Twenty-six patients were enrolled thus far in this ongoing study, with 25 subjects completing the ICG protocol in its entirety. One subject withdrew from the study in the pre-operative area due to IV site numbness and pain during the initial ICG infusion. Three subjects were excluded for bone sarcoma, and four subjects were excluded due to final pathology identifying a benign mass, despite fine needle aspiration biopsy exhibiting findings concerning sarcoma. As a result, 18 subjects with STS were included in this analysis (Table 1). The average age of subjects was 64.1 years old (range: 28–83 years old), with eight females and ten males. Seven subjects received pre-operative radiation therapy, and two subjects received pre-operative chemotherapy. Four of the subjects included had experienced LR and were presenting for additional surgery.

### 3.2. ICG Dosage, Duration, and Detection

The average ICG dose administered was 201.8 ± 52.7 mg, with an average infusion time of 37.6 ± 17.3 min (Table 2). Of note, we initially encountered calibration issues with the SPY-PHI camera intraoperatively, in part due to the tumor being highly oversaturated with very high ICG intensity. As a result, the weight-based dosing of ICG was reduced after 15 subjects were enrolled down to a dose of 2.0 mg/kg. Ten subjects included in this analysis thus received a dose of the original 2.5 mg/kg of ICG, with the remainder receiving 2.0 mg/kg dosing. This ICG dose was reduced with approval by our institution’s IRB, given the purpose of this proof-of-concept study. Any issues with calibrating the SPY-PHI camera were resolved after this dose adjustment was made. The ICG infusion was completed an average of 115.6 ± 80.4 min prior to the operation incision start time. To quantify the efficacy of distinguishing abnormal from normal tissue using ICG, a tumor background ratio was calculated with an average of 6.53 ± 2.61.

### 3.3. ICG Margin Comparison

The results of the ICG margin assessment, when compared with the surgeon’s impression and with the final pathology, are reported on a case-by-case basis in Table 3. Overall, ICG detected positive margins in 3 out of 18 cases, compared to 4 out of 18 based on surgeon impression and 8 out of 18 determined on final pathology. In 10 out of 18 cases (55.6% accuracy), ICG findings corresponded to final pathology results, with two true positives and eight true negatives. We calculated a sensitivity of 22.2%, specificity of 88.9%, with a positive predictive value of 66.7% based on these findings (Table 4). Comparatively, the surgeon’s impression matched the final pathology in 10 out of 18 cases. In two of the four cases where the surgeon’s impression anticipated a positive margin, the reason was for debulking or incomplete removal of the tumor with a planned positive margin. In both cases (Subjects 3 & 11, Table 3), the planned positive margin was in a patient with dedifferentiated liposarcoma, and ICG margins were negative, while permanent pathology margins were positive. ICG margins matched the surgeon’s blinded impression in 12 out of 18 cases. However, among the six cases where ICG margins did not match with the surgeon’s impression, two cases had positive margins found on ICG, which were subsequently confirmed on final pathology. Lastly, among the cases where false positives or false negatives occurred, the most common diagnoses based on pathology were dedifferentiated liposarcoma (two cases), myxofibrosarcoma (two), and spindle cell sarcoma (two).

**Figure 4:**
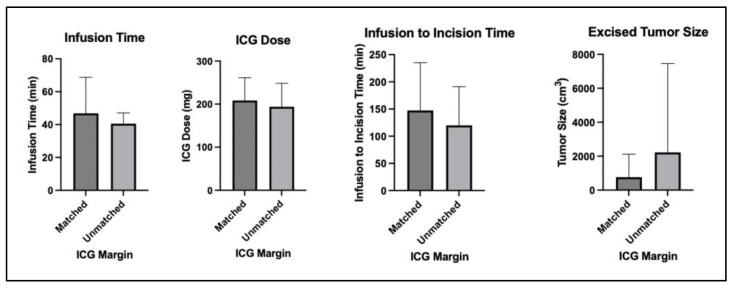
Bar graphs comparing infusion dose, infusion time, time to incision, and tumor size between cases where ICG margins matched final pathology and cases where ICG margins did not match final pathology.

A comparison was then performed between (1) cases where ICG results matched the final pathology and (2) cases where ICG results did not match the final pathology (Table 5). These matched versus unmatched groups were evaluated and compared in terms of infusion dose, infusion time, the time between infusion and first incision, and tumor size. There were no significant differences in these parameters (Figure 4). The groups were also compared in terms of prior chemotherapy, prior radiation, or local recurrence. No significant differences were observed in these parameters.

## 4. Discussion

The utility of ICG fluorescent imaging for intraoperative margin evaluation has been studied in breast, hepatic, gastrointestinal, urologic, thoracic, and ovarian cancers [19,22,29,30,34,35,36] and is now being explored as an adjunct to reduce LR rates in the treatment of STS [27,28]. Our study assessed the feasibility of ICG fluorescence imaging in visualizing STS margins and preliminarily evaluated if ICG intraoperative assessment could provide guidance in achieving negative surgical margins. Thus far in this ongoing study, we have observed that sufficient ICG uptake into STS tissue occurs such that abnormal tissue can be visualized using intraoperative fluorescence imaging. When effectively implemented, this technique can provide a real-time, intraoperative means for observing sarcoma tumor margins. Our findings corroborate Nicoli et al., who found ICG implementation in sarcoma surgery to be feasible, safe, and potentially useful as a guide for obtaining negative margins and hopefully decreasing LR rates [28].

From an ICG dosage and timing standpoint, we found that our strategy for ICG administration provided adequate visualization intraoperatively while still permitting the patient to present on the day of surgery for a pre-operative infusion. Various strategies for ICG administration exist within the literature, with the two most common strategies being ICG administration either 24 h preceding surgery [36,37,38] or immediately pre-operatively [22,23]. A time-dependent effect for adequate ICG deposition likely exists, as ICG pooling is believed to occur due to enhanced permeability and retention near areas of aberrant tissue architecture, such as in the setting of abnormal tumor vasculature [26]. Our rationale for administering ICG immediately pre-operatively was based on several considerations. Given that pre-operative administration sufficiently visualized breast and lung malignancies in prior studies, we wanted to observe if STS could also be visualized under similar circumstances. Although this technique still requires further validation, ICG administration pre-operatively compared to 24 h prior to surgery is advantageous from the standpoint of patient and system convenience. The next consideration of our ICG administration strategy involved appropriate dosing. Prior literature has administered ICG by weight-based dosing in a range from as low as 0.25 mg/kg to as high as 5 mg/kg, a range which has been safely tolerated by patients [23,30,36]. Newton et al. found that 2–3 mg/kg dosing effectively visualized non-lung malignancies, compared to 4–5 mg/kg dosing being superior for visualizing primary lung malignancies [39]. We initially selected a dose of 2.5 mg/kg and encountered oversaturation on several resected tumors. Due to the robust oversaturation encountered with this initial dose, we reduced our initial pre-operative weight-based dose to 2.0 mg/kg and observed the resolution of tumor oversaturation, and retained the ability to distinguish tumor from normal adjacent tissue. Brookes et al. performed a similar study where multiple dosing regimens were trialed in 39 sarcoma cases, including fixed doses between 25–100 mg as well as 1 mg/kg weight-based dosing either one day prior (16–24 h before surgery) or at time of anesthesia induction [27]. Although their study did not conclude an optimal dose or timing for ICG administration, they reported that infusion of ICG at the time of anesthesia induction resulted in more non-specific fluorescence compared to infusion the day prior to surgery. Abdelhafeez et al. also performed a similar study in pediatric solid tumors, including 23 sarcoma cases out of 55 total cases, who received 1.5 mg/kg of ICG administered the day prior to surgery [40]. Their study included multiple tumor types and noted 86% accuracy in distinguishing malignant tissue from normal tissue utilizing ICG fluorescent imaging intraoperatively. The study concluded that their timing and dosage protocols were appropriate for a pediatric population. Our study also found that infusion in the pre-operative setting sufficiently distinguished between normal and tumor tissue. We reported tumor background ratio (TBR), a measure for comparing fluorescent signal between tumor tissue and normal adjacent tissue (TBR = % Fluorescence of Tumor/% Fluorescence of Normal Tissue), as 6.53 ± 2.61 in our study. TBR, when measured ≥2.0, suggests an excellent ability to distinguish tumor tissue from normal adjacent tissue, and our finding is comparable to TBR reported in the breast cancer literature during ICG fluorescent imaging, with 1.8 ± 0.7 and 3.3 ± 1.7 reported in prior studies [29,30], as well as with 4.42 ± 2.91 reported within the pancreatic cancer literature [39]. In summary, it appears that several strategies for administering ICG are feasible for distinguishing fluorescence in abnormal tumor tissue, and our strategy successfully achieved a TBR that confers practical utility.

Despite our study effectively implementing ICG fluorescence imaging at our stated dosing and administration strategy, our study has not definitively demonstrated if this technique confers any meaningful benefit in predicting final pathology margins or if the incidence of LR would decrease as a result of this tool. The protocol we adapted from prior studies sets fluorescence in the tumor bed of ≥77% as indicative of a positive margin [29,30]. Using this threshold, we obtained a sensitivity of 22% and specificity of 89% for ICG-based margin evaluation compared to final pathology results. Notably, our study compared ICG margins to final pathology margins in a fashion where the operating surgeon was blinded to the ICG results intraoperatively. This strategy for assessing the efficacy of ICG-guided margin resection was distinct from Brookes et al.’s strategy [27,28], which provided the operating surgeon with full access to ICG imaging intraoperatively. The surgeon was then post-operatively surveyed on if they believed ICG helped expand or guide their final resection margin beyond their pre-operative plan based on MRI. In 11 cases in this 39-patient series, the surgeon noted ICG use provided guidance in performing further tissue resection to improve margins. Comparatively, we are investigating if fluorescent tissue concerning a positive ICG margin corresponds to a positive margin on final pathology. Presumably, utilizing ICG-guided resection without validating its accuracy in revealing positive margins could result in unintentional collateral damage from the over-resection of normal, uninvolved tissue. Over-resection for the purpose of obtaining negative margins does not come without potential risks, including wound complications, diminished functional status, and prolonged intraoperative time. Although we report a sensitivity of 22%, ICG had a specificity of 89% and therefore showed promise in improving a surgeon’s ability to evaluate margins intraoperatively. Among the six cases where ICG margins contrasted with the margin impressions made by our surgeons, two cases were ICG-positive with resultant positive margins on final pathology. In circumstances such as this, the use of ICG may indeed augment a skilled surgeon’s ability to identify and resect areas of unremoved tumor. Conversely, there may also be types of soft tissue sarcomas that are less amenable to ICG fluorescent imaging. Amongst our false positive or false negative cases, we found that two cases, each of dedifferentiated liposarcoma, myxofibrosarcoma, and spindle cell sarcoma, were represented. A larger sample size is required to fully test the utility of ICG fluorescent imaging in guiding surgical resection under different circumstances. In the final analysis, the LR rate is clearly a more meaningful outcome measure than margin status. Our ultimate goal is to determine if ICG can enable fewer LRs and thus improve patient outcomes.

The study we performed comes with several limitations, most notably the small sample size and heterogeneity of the patients enrolled thus far. The latter of these may be regarded as an advantage from a practical standpoint, as it embraces the complexity inherent to the care of STS patients. Indeed, STSs are an extremely heterogeneous group of related tumors, with >50 histological subtypes that may exhibit different responses to ICG as a function of the STS histologic subtype. There are also limitations to our ICG administration strategy. Every effort was made to control for the dosage and timing to be uniform. However, changes to our dosing protocol had to be made due to the oversaturation of tumor tissue, and the timing of infusion was dependent on the patient time of arrival, with some patients infused closer to their surgical incision time than others. This, too, may be ultimately seen as an advantage that acknowledges and respects the practical pressures of STS surgery.

Despite the limitations of this study, we believe it validates the notion that pre-operative administration of ICG is appropriate for visualizing STSs with fluorescence imaging to distinguish tumor tissue from normal tissue. The accuracy, sensitivity, specificity, and clinical relevance of ICG for intraoperative margin evaluation still warrant further investigation. We will continue to enroll sarcoma patients and hypothesize that a larger sample size will answer these important questions. In the future, developing a better understanding of the effects of tumor histologic subtype, infusion time, dosage, and neoadjuvant chemotherapy/radiation may help to improve this technique and decrease the incidence of LR.

## 5. Conclusions

This study demonstrates that the intraoperative evaluation of STS surgical margins with ICG fluorescence imaging is feasible. Although this study’s accuracy was greater than 50%, the ability of ICG margins to mirror final pathology margins warrants further investigation and validation. Determining appropriate dosing, timing, and settings in which ICG can effectively demarcate tumor margins should be the focus of future studies to improve the efficacy of this emerging technique in the hope of lowering LR rates.

## Figures and Tables

**Table 1:** Demographics for the study population of 18 patients undergoing resection of soft tissue sarcoma.

Demographics (*n* = 18)	
Age (years)	64.1 ± 14.1
Gender	8 female (44%), 10 male (56%)
Local Recurrence	4/18 (22%)
Prior Chemotherapy	2/18 (11%)
Prior Radiation	6/18 (33%)

**Table 2:** Reported average values for ICG dosage, timing of ICG infusion, as well as tumor background ratio calculated from intraoperative assessment footage.

ICG Dosing and Duration	
ICG Dose (mg)	201.8 ± 52.7
Infusion Duration (min)	37.6 ± 17.3
Time Between Infusion to Surgical Incision (min)	115.6 ± 80.4
Tumor Background Ratio	6.53 ± 2.61

**Table 3:** Soft tissue sarcoma margin assessment by case.

ICG Margin Case-by-Case Comparison				
Subject	Diagnosis	Margins (Surgeon)	Margins (ICG)	Margins (Pathology)
1	Pleomorphic liposarcoma	Negative	Positive	Positive
2	High grade round cell sarcoma	Negative	Positive	Negative
3	Dedifferentiated liposarcoma	Positive	Negative	Positive
4	Pleomorphic dermal sarcoma	Negative	Negative	Negative
5	Epithelioid inflammatory myofibroblastic sarcoma	Negative	Negative	Negative
6	Leiomyosarcoma	Negative	Negative	Negative
7	Sclerosing rhabdomyosarcoma	Negative	Negative	Negative
8	Spindle cell sarcoma	Negative	Negative	Positive
9	Pleomorphic sarcoma	Negative	Negative	Negative
10	Myxofibrosarcoma	Negative	Positive	Positive
11	Dedifferentiated liposarcoma	Positive	Negative	Positive
12	Myxofibrosarcoma	Negative	Negative	Positive
13	Spindle cell sarcoma	Negative	Negative	Positive
14	Synovial sarcoma	Negative	Negative	Negative
15	Dedifferentiated liposarcoma	Positive	Negative	Negative
16	Myxofibrosarcoma	Negative	Negative	Positive
17	Pleomorphic liposarcoma	Positive	Negative	Negative
18	Pleomorphic sarcoma	Negative	Negative	Positive

**Table 4:** Comparison of margin evaluation tools to final pathology results.

Sensitivity, Specificity, and Predictive Values Compared to Permanent Pathology. PPV, Positive Predictive Value; NPV, Negative Predictive Value				
Margin Evaluation	Sensitivity (%)	Specificity (%)	PPV (%)	NPV (%)
ICG	22.2	88.9	66.7	53.3
Surgeon Impression	22.2	77.8	50.0	50.0

**Table 5:** Comparison between matched and unmatched cases of ICG implementation. Continuous variables were compared using an unpaired Student’s *t*-test, and categorical variables were compared using a Chi-Square test.

Comparison between Cases Where ICG Margins Matched or Did Not Match Margin Descriptions on the Case’s Final Pathology Report			
	Matched (*n* = 10)	Unmatched (*n* = 8)	Significance (a = 0.05)
Infusion dose (mg)	208 ± 53	194 ± 55	*p* = 0.58
Infusion time (min)	46 ± 22	40 ± 6	*p* = 0.48
Infusion to incision time (min)	147 ± 88	120 ± 71	*p* = 0.50
Excised tumor size (cm^3^)	762 ± 1356	2220 ± 5237	*p* = 0.41
Prior chemotherapy	1/10	1/8	*p* = 0.93
Prior radiation	3/10	3/8	*p* = 0.89
Local recurrence	2/10	2/8	*p* = 0.79

## Data Availability

The data presented in this study are available on request from the corresponding author. The data are not publicly available due to privacy reasons and the ongoing nature of the study.
